# Mr. Ring, on Vaccine Inoculation

**Published:** 1801-04

**Authors:** John Ring

**Affiliations:** New Street, Hanover Square


### *To the Editors of the Medical and Phyfical Journal.*

GENTLEMEN,

LETTERS have been received from Dr. J. H. Marfhall, dated Malta, Dec. 18, 1800, in which he gives an account of his arriving at that place on the 1ft of the fame month, and commencing the practice of Vaccine Inoculation the next day, by inoculating feveral children in the Foundling Hofpital, who were proceeding through the difeafe in the moft favourable way.

Lord Keith and Sir Ralph Abercrombie have iffued general orders for the inoculation of the foldiers and failors under their command. Dr. Marfhall adds, "In all our experience we have not obferved a fingle deviation from the ufual mild and gentle appearance of the Cow-pox in England; nor has a fingle foldier or failor been prevented from doing his ordinary duty."

It is now, therefore, evident how little reafon there was for the alarm excited with fo much induftry, that our brave tars were to be made the victims of experiment. Previous to receiving this intelligence from Dr. Marfhall, I received from my nephew, Mr. J. Ring, an equally favourable account of the good fuccefs attending Cow-pock Inoculation at Chatham, which was communicated to him by the furgeon of the marine barracks.

This is the place to which all recruits are firft fent to be approved of by a general officer appointed by government, and where it is cuftomary to inoculate thofe who have not had the Small-pox as foon as they arrive, which has for fome time been done with the Cow-pox.

The numbers wanted have been fo great that the Cow-pock patients have not been entered on the fick lifts, confequently they were obliged to perform the fame duty as the others; which which they have hitherto done, without perceiving any inconvenience.

Since I wrote my former Obfervations on the cure of the Bronchocele, Mr. Pearfon informs me, that his experience in the treatment of the difeafe perfectly coincides with mine. He has frequently given large quantities of the fpongia ufta in other forms, for a confiderable time, to no purpofe; and afterwards effected a cure by adminiftering a fmaller quantity in the form of a lozenge.—As a proof of the mild and innocent nature of this remedy, he has given fome patients an ounce a day of that article without the leaft injury.

I readily agree with Mr. Moore, that the cafe he defcribes in your laft, was of an inflammatory kind; but think it ought to be confidered as a cafe of Phrenitis rather than Synocha. I beg leave to fuggeft, with due deference to the learned and ingenious author, that a ftout man, of a *florid complexion*, and a *full fanguine habit*, under the influence of *extreme terror*, with a *pain in his forehead*, *his face redder than ufual*, and *his eyes flightly inflamed*, with a *pulfe hard*, *ftrong*, *and regular*, might poffibly have borne the lofs of blood, on the fixth day, or on *any day* of the difeafe, not only with fafety, but even with advantage.

We are informed, that from the fixth day, when Mr. M. firft faw him, the difeafe increafed, though with occafional remiffions, for four days. The fkin was hot, the patient difpofed to conftipation; thirfty; fhewed no figns of naufea, or want of appetite, but fwallowed readily whatever was given him. On the tenth day he was quite furious, and could hardly be kept in bed, though ftrapped down, and reftrained in bed by two ftrong men.

That Mr. M. faved the life of his patient by a rigid adherence to the antiphlogiftic plan in every other refpect, inftead of treating the difeafe as a Typhus, and difpatching his patient by the affiftance of bark and wine, as is too much the fafhion, will, I prefume, be acknowledged by the moft refpectable part of the medical profeffion.

From the well-known candour and liberality of the author of the paper here commented on, whom I have the honour to call my friend, I doubt not but he will excufe the freedom of thefe remarks. I am, &c.

*New Street, Hanover Square,* JOHN RING.

*March* 19, 1801.

